# DDI-MuG: Multi-aspect graphs for drug-drug interaction extraction

**DOI:** 10.3389/fdgth.2023.1154133

**Published:** 2023-04-24

**Authors:** Jie Yang, Yihao Ding, Siqu Long, Josiah Poon, Soyeon Caren Han

**Affiliations:** ^1^ School of Computer Science, The University of Sydney, Sydney, NSW, Australia; ^2^ Department of Computer Science, University of Western Australia, Perth, WA, Australia

**Keywords:** drug-drug interactions, relation extraction, deep learning, multi-aspect graphs, graph neural network

## Abstract

**Introduction:**

Drug-drug interaction (DDI) may lead to adverse reactions in patients, thus it is important to extract such knowledge from biomedical texts. However, previously proposed approaches typically focus on capturing sentence-aspect information while ignoring valuable knowledge concerning the whole corpus. In this paper, we propose a Multi-aspect Graph-based DDI extraction model, named DDI-MuG.

**Methods:**

We first employ a bio-specific pre-trained language model to obtain the token contextualized representations. Then we use two graphs to get syntactic information from input instance and word co-occurrence information within the entire corpus, respectively. Finally, we combine the representations of drug entities and verb tokens for the final classification

**Results:**

To validate the effectiveness of the proposed model, we perform extensive experiments on two widely used DDI extraction dataset, DDIExtraction-2013 and TAC 2018. It is encouraging to see that our model outperforms all twelve state-of-the-art models.

**Discussion:**

In contrast to the majority of earlier models that rely on the black-box approach, our model enables visualization of crucial words and their interrelationships by utilizing edge information from two graphs. To the best of our knowledge, this is the first model that explores multi-aspect graphs to the DDI extraction task, and we hope it can establish a foundation for more robust multi-aspect works in the future.

## 1. Introduction

According to statistics from the U.S. Centers of Disease Control and Prevention, from 2015 to 2018, 48.6% of Americans used at least one prescription drug in 30 days.^1^ More seriously, 20% of the elderly took more than 10 drugs simultaneously (1). However, drug-drug interaction (DDI) may occur when patients take multiple drugs, resulting in reduced drug effectiveness or even, possibly, adverse drug reactions (ADRs) (2). Therefore, the study of DDI extraction can be considerably important to patients’ healthcare, as well as clinical research. Currently, a number of drug databases, such as DailyMed (3), TWOSIDES (4) and DrugBank (5) can be used for retrieving DDI knowledge directly. However, with the exponential growth in biomedical literature, huge amounts of the most current and valuable knowledge remain hidden in biomedical literature (1). Thus, the development of an automatic tool to extract DDI is an urgent need.

During the past few years, various deep learning-based approaches, such as (6–14) have been proposed to extract DDI knowledge. Recently, (15) proposed an Long Short-Term Memory(LSTM)-based RNN model with two distinct additional layers, i.e., bottom RNN and top RNN, for the DDI extraction. It is worth noting that compared with LSTM, Graph Neural Networks (GNNs) can better deal with complex structural knowledge. Based on this, Li and Ji (8) combined a Bio-specific BERT (16) and Graph Convolutional Network (GCN) (17) to capture contextualized representation together with syntactic knowledge. Shi et al. (13) adopted the Graph Attention Network (GAT) (18) on an enhanced dependency graph to obtain higher-level drug representations for DDI extraction. However, as examples in Table 1, all the previous models only pay attention to the sentence-aspect features and do not even exploit the corpus knowledge, which could cause essential clues to be overlooked.

**Table 1:** Summary of previous neural network-based models and our proposed model.

Model	Sentence (semantic)	Sentence (syntactic)	Corpus
AB-LSTM (19)	GloVe (20)	No	No
DCNN (6)	Order embedding (21)	No	No
ASDP-LSTM (7)	Word2Vec (22)	Dependency parse	No
RHCNN (23)	Bio-word emb. (24)	Dependency parse	No
GCNN-DDI (25)	Bio-word emb. (24)	Dependency parse	No
BERTChem-DDI (10)	BioBERT (26)	No	No
BERTDesc-DDI (11)	SciBERT (27)	No	No
**DDI-MuG** (Ours)	PubMedBERT(28)	Dependency parse	PMI

To alleviate the issues mentioned above, in this work, we propose a multi-aspect graphs-based DDI extraction model, DDI-MuG, which can make use of the information in both sentence and corpus aspects. First, we use PubMedBERT to obtain sentence semantic representation. We then apply a GCN with an average pooling layer to capture syntactic features from the input instance, and another GCN with average pooling is employed to model the word co-occurrence in the corpus level simultaneously. After that, attentive pooling is used to integrate and obtain the optimal feature from the output of PubMedBERT and both sentence-aspect and corpus-aspect graphs. Finally, we employ a fully connected neural network in the output layer for the classification. Our proposed model is evaluated on two benchmark datasets: DDIExtraction-2013 (29) and TAC 2018 corpora (30). Experimental results show that our proposed model improves the performance of DDI extraction effectively.

To recap, the main contributions of our work can be summarized as follows:

∙ We propose a novel neural model, named DDI-MuG, to exploit information from sentence-aspect and corpus-aspect graphs. As far as we know, this is the first model that utilizes multi-aspect graphs for the DDI extraction task.
∙ We explore the effectiveness of different components in DDI-MuG. Experimental results indicate that knowledge from multi-aspect graphs is complementary, and their effective combination can largely improve performance.
∙ We evaluate the proposed model on two benchmark datasets and achieve new state-of-the-art performance on both of them.

The rest of the paper is organized as follows. First, we introduce the background in Section 1. Then, several related works are introduced in Section 2. Next, in Section 3, we explain the framework in the proposed model in detail. We then describe the two benchmark datasets, evaluation metrics, and parameter setting in Section 4. Section 5 presents the experimental results and discussion, and finally, we conclude this work in Section 6.

## 2. Related works

Knowledge in many applications is exceedingly complex for a single-aspect network to learn robust representations. Multi-aspect networks have thus emerged naturally in different fields. Khan and Blumenstock (31) developed a multi-aspect GCNs model to consider different aspects of phone networks for poverty research. They employed subspace analysis and a manifold ranking procedure in order to merge multiple views and prune the graph, respectively. Liu et al. (32) first constructed semantic-based, syntactic-based, and sequential-based text graphs, and then utilized an inter-graph propagation to coordinate heterogeneous information among graphs. In order to exploit richer sources of graph edge information, Gong and Cheng (33) resorted to multi-dimensional edge weights to encode edge directions. Similarly, Huang et al. (34) used multi-dimensional edge weights to exploit multiple attributes, adapting the edge weights before entering into the next layer. In order to improve the prediction accuracy of social trust evaluation, Jiang et al. (35) assigned different attention coefficients to multi-aspect graphs in online social networks. Recently, Zhang et al. (36) constructed MA-GNNs, which utilize multiple aspect-aware graphs to improve recommendation performance. This model disentangles user preferences into different aspects and constructs multiple aspect-aware graphs to learn aspect-based user preferences.

## 3. Methods

**Figure 1:**
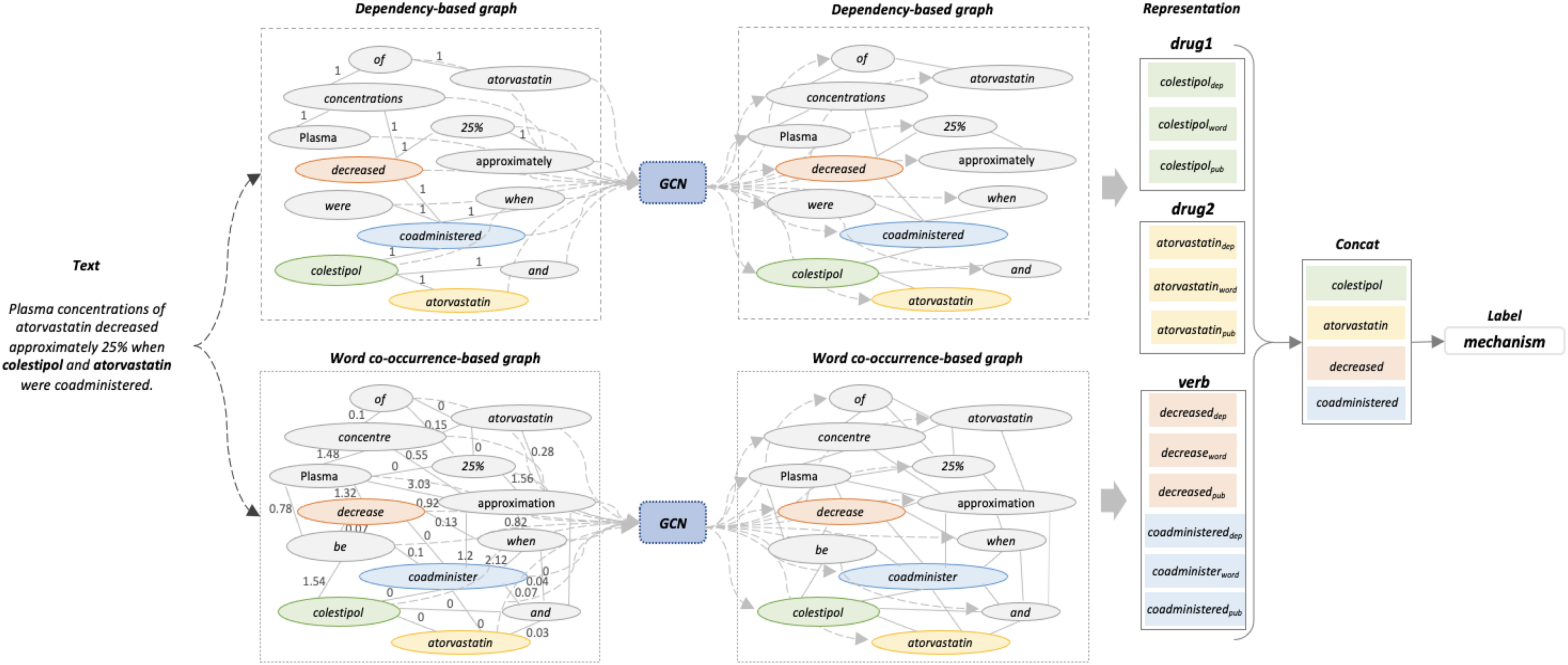
The proposed model architecture. This example is selected from DDIExtraction-2013 dataset. Two drugs are labelled in bold. As the space is limited, only part of the edges is shown in the word co-occurrence-based graph.

The architecture of the proposed model is illustrated in Figure 1. First, we obtain the contextual semantic representation of the input instances by PubMedBERT. Then, a sentence-aspect graph is constructed to encode the syntactic feature from the dependency path, while a corpus-aspect graph is used to explore word co-occurrence within the entire corpus. Based on the vocabulary and instances analysis, we find that the part-of-speech (POS) tag of words, especially words corresponding to verbs, might be helpful for the final representation. Therefore, we subsequently feed the representations of verbs and drug entities from PubMedBERT, together with the two graphs, into an attentive pooling layer to distinguish important features from all representations. Finally, a fully connected layer with softmax is employed to perform the classification. The process is described in the following subsections in detail.

### 3.1. Encoding sentences with PubMedBERT

PubMedBERT was pre-trained on 14 million biomedical abstracts with 3.2 billion words from scratch. Given an input sentence $S=[w_{1},w_{2},\ldots ,w_{n},\ldots ,w_{t}]$ with drug entities $d_{1}$ and $d_{2}$, we convert each word $w_{i}$ into word pieces and then feed them into PubMedBERT. After the PubMedBERT calculation, we employ average pooling to aggregate vectorial representations of word pieces as the word representations. We denote the two drugs and verbs representations by $drug1_{\;pub}$, $drug2_{\;pub}$, and $verbs_{\;pub}$ respectively.

### 3.2. Graph construction

Considering a graph with *n* nodes, the node *i* at the lth layer is updated based on the representation of all neighbourhood nodes in the (l−1)th layer as follows:

(1) $$H^{l}=\sigma (\hat{A}H^{l-1}W^{l})$$

Here, $\hat{A}={\tilde{D}}^{-1/2}\tilde{A}{\tilde{D}}^{1/2}$ represents the normalized adjacency matrix, and $\tilde{A}=A+I$ is the adjacency matrix with added self-connections. $\tilde{D}$ is the diagonal node degree matrix with $\tilde{D}(i,i)=\sum_{\;j}\tilde{A}(i,j)$. $H^{l}\in R^{n*d_{l}}$ is the node embedding matrix at the *l*th layer, *n* is the number of nodes, $d_{l}$ indicates the dimension of the node features. Finally, $W^{l}\in R^{d_{l}*d_{l+1}}$ denotes a layer-specific trainable weight matrix, and σ is a nonlinear function.

For each input instance, we encode a dependency graph from the current instance and a word co-occurrence over the entire corpus.

#### 3.2.1. Sentence-aspect dependency graph

**Figure 2:**
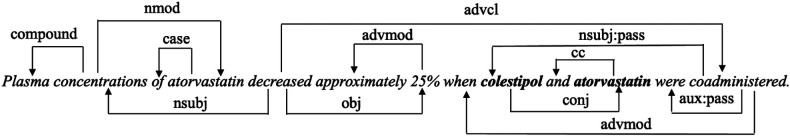
An example of dependency relation. Two drugs are labelled in bold.

Dependency parser is widely used in relation classification tasks with the aim of exploring the syntactic information of sentences. We apply the Stanford dependency parser (37) to extract dependency syntactic information. Figure 2 shows the dependency relation of the input text in Figure 1. The connection from *coadministered* to *colestipol* means that *coadministered* is the head word of *colestipol*, and *“nsubjpass”* denotes the *“passive nominal subject”* dependency relation between the two words. We use the word embedding from PubMedBERT as the initial node representations, and set edge weights as 0 or 1 to indicate if two nodes are connected in the dependency path.

Let the node representations in *l*th layer of the dependency graph be $M^{l}$. We apply two graph convolutional layers to update each node, thus the updated $M^{2}$ is expressed as follows:

(2) $$M^{2}=\sigma (\hat{A}M^{1}W^{2})$$

Then, an average pooling layer is applied to get the syntactic-based sentence embedding. Let $d_{1},d_{2},\ldots ,d_{n},\ldots ,d_{t}$ be the updated node representations obtained from graph convolutional layers, the output of dependency graph, $G_{Dep}$, is shown as:

(3) $$G_{Dep}=\underset{1\leq i\leq t}{avg}[d_{i}]$$

We denote the outputs of drug and verbs representations as $drug1_{dep}$, $drug2_{dep}$, and $verbs_{dep}$, respectively.

#### 3.2.2. Corpus-aspect word co-occurrence graph

Information on the co-occurrence of words indicates the connection between them, such as whether they form as a common phrase or provide clues for classification tasks. Firstly, we first lemmatize each word with Natural Language Toolkit (NLTK).^2^ Then we connect all word pairs in the graph, and employ point-wise mutual information (PMI) (38), a word associations measure, to store the word correlation information as an edge weight as follows:

(4) $$A_{ij}=\left\{ \begin{array}{cc} 1, & i=j\\ PMI(i,j), & i\neq j,PMI(i,j)>0\\ 0, & i\neq j,PMI(i,j)\leq 0 \end{array}\right.$$

The PMI between any two words is calculated as:

(5) $$PMI(i,j)=\log\frac{\;p(i,j)}{p(i)p(j)},$$

(6) $$\;p(i,j)=\frac{\#W(i,j)}{\#W},\;\;p(i)=\frac{\#W(i)}{\#W}.$$

where i,j are words, #W(i,j) is the number of examples in a fixed sliding window that contains both words, #W(i) is the number of instances in the sliding window that contain word *i*, and #W is the total number of sliding windows. It is worth noting that the entire input sentence is set as the sliding window. Suppose there are 31,738 instances in the corpus, and the word of *“decrease”* and *“coadminister”* appear 1,821 and 953 times, respectively, and that they occur 27 times together in the whole corpus. Based on Formula 5 to 6, the PMI between these two words is -4.8. A positive PMI value corresponds to a high correlation between two words, while a negative value means that the two words have a small probability or no probability of occurrence. When two words have a negative PMI value, we view them as non-co-occurring and set their edge weight as 0.

Suppose the node representations in *l*th layer is $N^{l}$. Similar to the dependency graph, the updated $N^{2}$ is shown as:

(7) $$N^{2}=\sigma (\hat{A}N^{1}W^{2})$$

After an average pooling layer was utilized to get the word co-occurrence-based embedding, the $G_{Word}$ graph is expressed as:

(8) $$G_{Word}=\underset{1\leq i\leq t}{avg}[w_{i}]$$

where $w_{i}$ is the updated *l*th node representation from graph convolutional layers.

Drug and verbs representations, denotes by $drug1_{word}$, $drug2_{word}$, and $verbs_{word}$, are extracted from $G_{Word}$ and used as input for the next layer.

### 3.3. Attentive pooling layer

So far, given two drug entities and verbs, we have obtained rich feature representations from PubMedBERT and two graphs. As each instance has a different number of verbs, we apply an attentive pooling to get a fixed-length representation for verbs. In detail, this pooling mechanism computes the weights of feature vectors by using an attention mechanism, allowing it to learn the most significant feature effectively. Let $A_{drug1}$ and $A_{drug2}$ be the combined representation of drug entities from PubMedBERT and the two graphs, and $A_{verbs}$ be the corresponding verbs representation:

(9) $$A_{drug1}=[drug1_{\;pub};drug1_{dep};drug1_{word}]$$

(10) $$A_{drug2}=[drug2_{\;pub};drug2_{dep};drug2_{word}]$$

(11) $$A_{verbs}=[verbs_{\;pub};verbs_{dep};verbs_{word}]$$

where [;] denotes concatenation. These three representations are fed into the attentive pooling layer separately as follows:

(12) $$H_{drug1}=tanh(A_{drug1})$$

(13) $$\alpha =Softmax(w^{a}H_{drug1})$$

(14) $$z_{drug1}=\alpha A_{drug1}$$

where $w^{a}$ is the learning parameter, α is the attention weights. $z_{drug1}$, $z_{drug2}$ and $z_{verbs}$ are the representation of the two drugs and verbs as the output of the attentive pooling layer.

### 3.4. Fully connected and softmax layer

In this layer, the updated representation of two drugs and verbs are concatenated as $z_{total}$, and a nonlinear activation function *tanh* is then applied over $z_{total}$ into a fully connected layer. Finally, we deploy a softmax with a dropout layer to get the probability score for each class. The process is expressed as follows:

(15) $$z_{total^{\prime }}=tanh(z_{total})$$

(16) $$p(y\;|\;x)=Softmax(W^{s}z_{total^{\prime }}+b^{s})$$

where $z_{total^{\prime }}$ is the output of the fully connected layer, $W^{s}$ and $b^{s}$ are the softmax matrix and the bias parameter, respectively.

## 4. Experiments

In our experiments, two public DDI extraction corpora, i.e., DDIExtraction-2013 and TAC 2018, were used to evaluate the proposed model. This section introduces the two corpora in detail and then presents the evaluation metrics and parameters setting.

### 4.1. DDIExtraction-2013 dataset

We obtained the corpus from the challenge SemEval-2013 Task 9 (39). This corpus is the major dataset that can be used to evaluate and compare the performance of DDI extraction models. It contains manually annotated sentences from 175 abstracts in MedLine,,^3^ and 730 abstracts in DrugBank.^4^ There are four kinds of positive interaction types: *Advice, Effect, Mechanism, Int*. If the two drugs are unrelated, their relations are labelled as *Negative*. The definitions of the five types are as follows:

∙ **Advice**: a recommendation or advice regarding the simultaneous use of two drugs is described between two drugs.
∙ **Effect**: an effect or a pharmacodynamic mechanism is described between two drugs.
∙ **Mechanism**: a pharmacokinetic mechanism is described between two drugs.
∙ **Int**: a DDI occurs between two drugs, but no additional information is provided.
∙ **Negative**: there is no interaction between two drugs.

The original corpus suffers from a serious data imbalance problem. For example, the ratio of *Int* to *Negative* instances in the training set is 1:123.7, which heightens the difficulty of classifying drug pairs that hold *Int* relations, and continually affects the overall performance. To alleviate this data imbalance issue, many negative examples are filtered out in earlier studies, e.g., (2, 6, 19, 40–42). To ensure that the experimental results can be compared fairly with other baseline models, we adopted three rules in (6) to remove negative instances:

∙ If both drugs have the same name, remove the corresponding instances. The assumption is that drug will not interact with itself.
∙ If one drug is a particular case or an abbreviation of the other, filter out the corresponding instances. Several patterns, such as *“DRUG-A (DRUG-B)”* and *“DRUG-A such as DRUG-B”*, are used to identify such cases.
∙ If both drugs appear in the same coordinate structure, filter out the corresponding instances. Also, we use some pre-defined patterns, like *“DRUG-A, $(DRUG-N{)}^{+}$, DRUG-B”*, to filter out such instances.

Table 2 summarizes the statistics and divisions of this corpora.

**Table 2:** The statistics of DDIExtraction-2013 corpus.

		Training		Test	
		Original	Filtered	Original	Filtered
Positive	Advice	826	824	221	221
	Effect	1,687	1,676	360	358
	Mechanism	1,319	1,309	302	301
	Int	188	187	96	96
Negative		23,772	19,342	4,737	3,896
Overall		27,792	23,338	5,716	4,872

### 4.2. TAC 2018 corpus

One of the tasks in “Drug-Drug Interaction Extraction from Drug Labels” track of the Text Analysis Conference (TAC) 2018^5^ was to detect and extract DDIs from structured product labellings (SPLs). The organizers provided a set of 22 SPLs for training (Training-22). Two other datasets containing 57 and 66 SPLs were provided as test sets. The organizers also provided an additional 180 SPLs (NLM-180) to supplement the training set. Interactions in this corpus are classified into one of the following three types:

∙ **Pharmacokinetic**: This type includes phrases that demonstrate changes in physiological functions (30), such as *decrease exposure, increased bioavailability*.
∙ **Pharmacodynamic**: This type includes phrases that describe the effects of the drugs, e.g., *blood pressure lowering*.
∙ **Unspecified**: This type corresponds to caution phrases, e.g., *avoid use*.

As the original corpus is in XML format, we use the dataset in the KLncLSTMsentClf model (43) to train and evaluate our proposed model. In total, we obtain 6,436 training sentences by merging the training-22 and NLM-180 corpora. The two test sets contain 8,205 and 4,256 sentences, respectively.

### 4.3. Evaluation metrics

*precision(P), recall(R)* and *F-score(F)* are the major evaluation metrics in the DDI extraction task. In this paper, we adopt the standard micro-average *precision, recall* and *F-score* to evaluate the performance, and the formulas are listed as follows:

(17) $$Precision=\frac{TP}{\left(TP+FP\right)},$$

(18) $$Recall=\frac{TP}{\left(TP+FN\right)},$$

(19) $$\text{F-score}=\frac{2*P*R}{\left(P+R\right)}.$$

TP (true positive) represents the number of correctly classified positive instances, FP (false positive) denotes the number of negative instances that are misclassified as positive instances, and FN (false negative) is the number of positive instances that are misclassified as negative ones.

### 4.4. Parameters setting

In our experiment, PyTorch library (44) is used as the computational framework. As there is no development or validation set in the original corpus, we randomly select 20% of the training dataset as the validation set to adjust the model parameters and the remaining 80% as the training set. The parameters used are shown as follows:

∙ Maximal length n=128.
∙ Embedding size of PubMedBERT $m_{1}=768$.
∙ Hidden layer dimension of dependency and co-occurrence graph $m_{2}$ & $m_{3}=200$.
∙ Mini-batch size = 32.
∙ Dropout rate p=0.1.
∙ Learning rage lr=0.0001.
∙ Number of epoch = 10.

## 5. Results and discussion

### 5.1. Results on DDIExtraction-2013

#### 5.1.1. Comparison with baseline methods

We compare the performance of our DDI-MuG with 11 baseline methods. The comparison results of different models are shown in Table 3. The highest value is labelled in bold, and the second highest value is marked underline. In general, deep neural network-based approaches achieve better performance than statistical ML-based methods. It demonstrates the capability and potential of utilizing neural network in DDI extraction tasks. A notable exception is that the F1-score of SVM-DDI (40) is slightly higher than the AB-LSTM model (19). This might be due to SVM-DDI (40) benefiting from rich and complex lexical and syntactic handcraft features. It can be seen that our DDI-MuG obtains the best overall performances in view of precision and F1 score. In terms of the performances for all four types, DDI-MuG performs best on *Advice*, *Mechanism* and *Int*, and obtain the second best performance on *Effect*. It is worth noting that all methods achieve relatively low performance on *Int*. This discrepancy might be caused by the insufficient training samples of *Int*, which leads to these models to be underfitting.

**Table 3:** Performance comparisons on DDIExtraction-2013 Corpus. The highest value is labelled in bold, and the second highest value is marked underline.

Methods	Breakdown F1				Overall performance		
	Advice	Effect	Mechanism	Int	Precision	Recall	F1
*Statistical ML-based methods*							
UTurKu (45)	0.630	0.600	0.582	0.507	0.732	0.499	0.594
WBI (46)	0.632	0.610	0.618	0.510	0.642	0.579	0.609
FBK-irst (47)	0.692	0.628	0.679	0.547	0.646	0.656	0.651
SVM-DDI (40)	0.725	0.662	0.693	0.483	–	–	0.670
*Deep neural network-based methods*							
AB-LSTM (19)	0.697	0.683	0.681	0.542	0.678	0.659	0.669
DCNN (6)	0.777	0.693	0.702	0.464	0.757	0.647	0.698
Joint AB-LSTM (19)	0.794	0.676	0.763	0.431	0.734	0.697	0.715
ASDP-LSTM (7)	0.803	0.718	0.740	0.543	0.741	0.718	0.729
RHCNN (23)	0.805	0.734	0.782	0.589	0.773	0.737	0.754
GCNN-DDI (25)	0.835	0.758	0.794	0.514	0.801	0.740	0.770
DREAM (13)	0.848	0.761	0.816	0.551	0.823	0.747	0.783
*Our methods*							
DDI-MuG(with word. graph)	0.893	0.812	0.871	0.599	0.868	0.805	0.835
DDI-MuG(with dep. graph)	0.900	**0.826**	0.865	0.583	0.842	**0.835**	0.839
DDI-MuG	**0.907**	0.823	**0.893**	**0.606**	**0.870**	0.824	**0.847**

Then, we find the contributions of multi-aspect graphs to the proposed model. By removing in turn the sentence-aspect dependency graph and corpus-aspect word co-occurrence graph, our method reduces to DDI-MuG(with word. graph) and DDI-MuG(with dep. graph), respectively. From Table 3, we can see that the F1-score of DDI-MuG(with dep. graph) is higher than the F1-score of DDI-MuG(with word. graph), which proves that the syntactic features are indeed valuable for identifying the interaction relation between two drugs. Overall, it can be seen that the F1-score of DDI-MuG surpass the DDI-MuG(with word. graph) and DDI-MuG(with dep. graph) by 0.012 and 0.008, separately. This indicates that multi-aspect graphs are complementary to each other and together can serve as an appropriate supplement to contextual information.

#### 5.1.2. Impact of pre-trained embedding

To evaluate the efficiency of the pre-trained language model, we conduct experiments of replacing PubMedBERT with other similar models. As shown in Table 4, the four bio-specific models, i.e., BioBERT, SciBERT, ouBioBERT (48), and PubMedBERT, led to improvement over standard BERT. DDI-MuG by PubMedBERT achieves the best result for the reason that it was pre-trained on biomedical texts from scratch.

**Table 4:** The effect of pre-trained embedding. The highest value is labelled in bold.

Pre-trained embedding	P	R	f F1
DDI-MuG(by BERT)	0.801	0.790	0.795
DDI-MuG(by BioBERT)	0.843	0.816	0.829
DDI-MuG(by SciBERT)	0.839	0.825	0.832
DDI-MuG(by ouBioBERT)	0.850	**0.826**	0.838
DDI-MuG(by PubMedBERT)	**0.870**	0.824	**0.847**

#### 5.1.3. Error analysis

In addition, to present the above achievements, it is necessary to discuss the limitations of our approach. One common type of error is that the four kinds of positive instances are often misclassified as negative instances. This is due to the imbalanced data that small instance categories are misclassified as large instance categories. There is another notable error that 34.4% of *Int* type instances are misclassified as *Effect* type. This is because some *Int* instances have similar semantics to *Effect* instances. For example, in the following two instances:

∙ *“**arbiturates** may decrease the effectiveness of oral contraceptives, certain antibiotics, quinidine, **theophylline**, corticosteroids, anticoagulants, and beta blockers.”*
∙ *“**sulfoxone** may increase the effects of **barbiturates**, tolbutamide, and uricosurics.”*

The words *decrease* and *increase* are the clues for identifying interactions in the two semantically close sentences. However, the first instance belongs to the *Int* type, while the second belongs to *Effect*. The number of *Int* instances is far smaller than the number of *Effect* instances, which also leads to the occurrence of this kind of mistake.

#### 5.1.4. Are verb representations really helpful?

In our previous vocabulary and instances analysis, we found that in the DDIExtraction-2013 corpus, when instances contain the words *inhibit, increased, decreased*, there is a great possibility that the drug pair has the *Mechanism* relation. On the other hand, when instances contain *avoided, recommended* or *administered*, the drug pair is likely to have the *Advice* relation.

Thus, to further investigate how the verbs are important for the final classification, we studied the effect of extracting DDI only from the drug information without using the verbs knowledge. Table 5 shows the comparison of the performance with and without the verb information. This result indicates verb representation can serve as a supplement to improve the model performance.

**Table 5:** The comparison of with or without verbs information. The highest value is labelled in bold.

	Precision	Recall	F-score
DDI-MuG(drug-only)	0.863	0.823	0.843
DDI-MuG(all)	**0.870**	**0.824**	**0.847**

### 5.2. Results on TAC 2018

#### 5.2.1. Comparison with baseline model

Since we use the same dataset as KLncLSTMsentClf (43), we view it as the baseline model. From Table 6, we can see that our proposed model achieves better results in both two test sets, which indicates the transferability of our proposed model.

**Table 6:** Comparison with baseline models on the TAC 2018 corpus. The highest value is labelled in bold.

Dataset	Model	P	R	F1
Test1	KLncLSTMsentClf	0.470	0.620	0.530
Test1	DDI-MuG(with word. graph)	0.717	0.712	0.715
Test1	DDI-MuG(with dep. graph)	0.688	0.718	0.703
Test1	DDI-MuG(all)	**0.721**	**0.728**	**0.723**
Test2	KLncLSTMsentClf	0.490	0.670	0.567
Test2	DDI-MuG(with word. graph)	0.710	0.726	0.718
Test2	DDI-MuG(with dep. graph)	0.713	0.730	0.721
Test2	DDI-MuG(all)	**0.717**	**0.743**	**0.729**

## 6. Conclusions

In this paper, we propose DDI-MuG, a novel multi-aspect graphs framework for DDI extraction tasks. Concretely, a bio-specific pre-trained language model, PubMedBERT, is first employed to encode the context information of each word from the aspect of sentence semantic information. Then, two graphs are utilized to explore sentence syntactic and corpus word co-occurrence information, respectively. After that, an attentive pooling mechanism is employed to update the representations of drug entities and verbs. Finally, by feeding the concatenated representation of the two drugs and verbs into a fully connected and softmax classifier, the interaction between the two drugs is obtained. Extensive comparison experiments with baseline models on two public datasets verify the effectiveness of multi-aspect graphs in the DDI extraction task.

In addition, Most previous models are based on the *black-box* concept that makes the prediction without showing how the model did so. However, with our proposed model, we can visualise the important words and its word-word relationship of the final classification by using the edge information in both dependency and co-occurrence graphs.

For future work, there are at least two directions that could be considered. Firstly, the performance on categories with small training samples, like *Int* in the DDIExtraction-2013 corpora, is unsatisfactory. The solution of contrastive learning can be explored. Secondly, drug knowledge from external databases could be integrated with the architecture for richer drug representations.

## Data Availability

Publicly available datasets were analyzed in this study. This data can be found here: https://github.com/zhangyijia1979/hierarchical-RNNs-model-for-DDI-extraction/tree/master/DDIextraction2013.

## Footnotes

^1^ https://www.cdc.gov/nchs/data/hus/2019/039-508.pdf

^2^ https://www.nltk.org/

^3^ https://www.nlm.nih.gov/bsd/medline.html

^4^ https://go.drugbank.com/

^5^ https://tac.nist.gov/2018/
